# Editorial: The genetic history of human populations along the ancient silk road

**DOI:** 10.3389/fgene.2023.1130104

**Published:** 2023-01-24

**Authors:** Xin Chang, Horolma Pamjav, Maxat Zhabagin, Shaoqing Wen

**Affiliations:** ^1^ Institute of Archaeological Science, Fudan University, Shanghai, China; ^2^ Hungarian Institute for Forensic Sciences, Institute of Forensic Genetics, Budapest, Hungary; ^3^ National Center for Biotechnology, Astana, Kazakhstan; ^4^ MOE Laboratory for National Development and Intelligent Governance, Fudan University, Shanghai, China; ^5^ Center for the Belt and Road Archaeology and Ancient Civilizations, Fudan University, Shanghai, China

**Keywords:** Silk Road, STRs (short tandem repeats), SNPs (single nucleotide polymorphism), ancient DNA (aDNA), DNA sequencing, DNA genotyping, mtDNA, Y chromosomal DNA

## Introduction

The Silk Road, a historical network of interlinking trade routes across the Afro-Eurasian landmass, was of great importance to the transport of peoples, goods, and ideas between the East and the West. Although its main use was for importing silk from China, traders moving in the opposite direction carried to Central China jewelry, glassware, and other exotic goods from the Mediterranean, jade from Khotan, and horses and furs from the nomads of the Steppe. In historical records, communication between China and Central Asia has been unbroken ever since the opening of the Silk Road in the Han Dynasty. However, relics unearthed from archaeological sites indicate that communication between people along the Silk Road began during the Bronze Age. The Silk Road brought together the achievements of the different peoples of Eurasia to advance the Old World as a whole.

Ethnic groups with different religions, cultures and customs inhabited the Ancient Silk Road and experienced complex histories. Patterns in genetic variation between individuals can tell us about the population history of these groups. In recent years, using relatively direct means of studying ancient samples through osseous material, alongside indirect means of analyzing the genomes of modern populations, demographic history—migrations, expansions and colonizing events - have been progressively revealed in numerous genetic studies of early human populations. However, until the present, the origins of the populations along the Ancient Silk Road and relationships have been examined in far less detail.

In this special editorial, we collected 15 genetic investigations involving the populations living along or related to the Silk Road from ancient times to the present day. These studies approach academic and public Research Topic (population origins, differentiation, and admixture) of the targeted populations through genome-wide sequencing (Kairov et al.; Allen et al.; Guarino-Vignon et al.) or microarray technologies (Ma et al.; Wang et al.; Zhang et al.), or various kinds of markers, including mtDNA (Ning et al.; Xue et al.; Xiong et al.; Xiong et al.; Cardinali et al.), Y chromosome (Chen et al.; Khussainova et al.), forensic STRs (Chen et al.; Adnan et al.; Khussainova et al.), SNPs (Chen et al.; Ma et al.; Wang et al.; Zhang et al.; Xiong et al.), and InDels data (Fan et al.) For example, a novel 6-dye direct and multiplex PCR-CE-based typing system has now been validated and could be considered as a reliable tool for human identification and intercontinental population differentiation (Fan et al.)

## Eastern end

The eastern end of the Silk Road lies on the middle reaches of the Yellow River. The Shimao site in Shaanxi Province is an important Neolithic archaeological culture in this area. To further reconstruct the genetic structure of Silk Road-related populations, it is necessary to understand the genetic compositions of such early local populations as these. The Shimao population showed a mostly local origin and showed a maternal affinity with Taosi site, a Longshan culture population in the middle Yellow River valley (Xue et al.).

The Hexi corridor connected the Central Plains with the Western Regions (present-day Xinjiang) and was an integral component of the eastern section of Silk Road. Heishuiguo site and Foyemiao site lie in the central and western portion of the Hexi corridor, respectively (Xiong et al.; Xiong et al.). The former site dates back to the Han Dynasty (118BCE-191CE) and the latter to the Wei - Jin Dynasties and Sui and Tang Dynasties (220CE- 907CE). For the two sites, from the paternal Y-chromosome perspective, all male individuals showed the Sino-Tibetan speaking origin of Yellow River-related populations, while Foyemiao samples demonstrated a higher proportion of an Altaic speaking and North Eurasian ancestral component, alongside a small proportion of southern East Asian ancestry. From the maternal perspective, female Heishuiguo individuals showed a northeast Asian origin and revealed a sex-biased migration from the middle and lower reaches of Yellow River to Hexi corridor. This was consistent with evidence in historical records, especially unearthed slips (Ge et al., 1997; Wen et al., 2004a). The female Foyemiao samples had a similar genetic structure with males. The genetic difference between Heishuiguo and Foyemiao populations reflected the distinct genetic diversity of the spatial-temporal Hexi Corridor. Furthermore, combined with multidisciplinary evidence, these two studies demonstrated the impact of sex-biased migration on the Hexi Corridor, providing a reference for other fields.

## The Western regions

Population admixture also can be observed in the Western Regions (present Xinjiang), where individuals at the Xiabandi reveal genetic flow from Central Asian populations and thus the influence of Middle and Late Bronze Age steppe pastoral cultures (Ning et al.) Another site, Shichengzi, a Han Dynasty agricultural garrison, also located in ancient Xinjiang, has revealed to us genetic makeup of a Frontier population in early imperial China (Allen et al.). Archaeogenomics at Shichengzi has revealed two subgroups with East Asian origin and Northeast Asian origin, respectively, occupying a single burial space. Interestingly, stable isotope analysis showed that dietary patterns among site inhabitants could be split among agro-pastoral and agricultural groups. Considering ancient DNA and stable isotope evidence together, it has been argued that Northeast Asian origins of Altaic pastoralists and East Asian origins of Han agriculturalists lived together in the Shichengzi military outpost.

## The northern steppe

The Altaic language family is divided into Mongolic, Tungusic and Turkic language groups. Altaic speaking nomads played an important role in shaping the northern steppe Silk Road. However, their history is often only recorded sporadically in ancient writings of surrounding civilizations. In this special edition, the genetic history of Uyghur (Adnan et al.), Kazakh (Kairov et al.; Adnan et al.; Khussainova et al.), Hui (Adnan et al.; Chen et al.; Ma et al.), Dongxiang (Ma et al.), Bonan (Ma et al.), Yugur (Ma et al.), Salar (Kairov et al.; Ma et al.), Mongol (Cardinali et al.), and Manchu (Zhang et al.) groups have been investigated using genetic markers and/or genome sequencing at the population level. Generally speaking, population from the same language group exhibit a closer genetic affinity. Furthermore, the Turkic-speaking population from China and Kazakhstan both present a closer genetic relationship to Central Asian populations, while Hui and Tungusic-speaking populations had a significant admixture with Han Chinese. Mongolian’ mitochondrial genomes show a dominant East Asian related ancestry, an outcome of Bronze Age events and Mongol Empire expansion along the Silk Road.

## Southern tea and horse ancient road

In addition to the northern steppe Silk Road, the Tea and Horse Ancient Road or South Silk Road were another part of the ancient Silk Road. Beginning at approximately 1200 years ago (Tang Dynasty), the Tea and Horse Ancient Road emerged as a famous caravan road system for tea, salt and horse trading in Southwest China. Guizhou Province, located near the Tea and Horse Ancient Road has been documented as a critical depository of substantial sociocultural, genetic, and linguistic diversity for studying southern Silk Road related populations. The southern Han constitute the majority ethnic group within Guizhou. Wang et al. found that the Guizhou Han were in turn a mixed population with shared excess ancestry with Longshan-culture-related middle Yellow River populations. Interestingly, Guizhou Han reveal significant genetic differentiation with geographically neighboring southern ethnic groups, such as the suspected descendants of Pengtoushan, Gaomiao, Daxi, Qujialing, Shijiahe, and other archaeological cultures (Wang et al.).

## Western end

At the western end of the Silk Road, Guarino-Vignon et al. found that a bronze age individual from Oxus Civilization (or Bactrio-Margian Archaeological Complex, BMAC) at the Ulug-depe site in Turkmenistan, shared genetic affinity with a local BMAC population, and further revealed that modern Central Asian Indo-Iranian–speaking populations primarily harbored ancient BMAC related ancestry (Guarino-Vignon et al.). The use of recent aboriginal paleogenomes, combined with the genomes of related modern humans, will help us to understand further details of the genetic history of regional populations.

## Conclusion

In summary, this special edition focuses on the population history along the Silk Road, covering the eastern limits of the Silk Road, the Western Regions, the northern steppe, and southern Tea and Horse Ancient Road, and western end. The genetic diversity and population structure of modern populations in these regions are dissected using ancient forensic markers. Moreover, combined with modern human DNA data, ancient DNA researches discuss the formation of targeted populations in greater detail. In future work, fine temporal-spatial scales using enlarged sample sizes should be considered when outlining changes in population dynamics along the ancient Silk Road.
